# Aerosol Therapy in Asthma–Why We Are Failing Our Patients and How We Can Do Better

**DOI:** 10.3389/fped.2020.00305

**Published:** 2020-06-11

**Authors:** Robert W. Morton, Heather E. Elphick, Vanessa Craven, Michael D. Shields, Lesley Kennedy

**Affiliations:** ^1^Sheffield Children's Hospital, Sheffield, United Kingdom; ^2^Queen's University Belfast, Belfast, United Kingdom; ^3^Royal Belfast Hospital for Sick Children, Belfast, United Kingdom

**Keywords:** asthma, child, poor control, inhaler technique, adherence, preventer therapy

## Abstract

In order for inhaled corticosteroids to be delivered adequately to the airways they require patients to take them regularly using an effective technique. Patients often have a poor inhaler technique, and this has been shown to result in sub-optimal asthma control. It is important for all clinicians prescribing inhaled medication to be experienced in the correct technique, and take time to train children so that they have mastered corrected inhaler technique. Using Teach to Goal or teach back methodology is a simple and effective way to provide this in the clinic setting. More than one training session is typically needed before children can master correct inhaler technique. Adherence to inhaled therapy has been shown to be sub-optimal in pediatric populations, with studies showing an average rate of around 50%. Subjective methods of measuring adherence have been shown to be inaccurate and overestimate rates. The advent of new technology has allowed adherence rates to be measured electronically, and it has been shown that regular feedback of these data can be effective at improving asthma control. New mobile apps and smart technology aim to engage patients and families with their asthma care. Effective use of these apps in collaboration with health care professionals has a vast potential to improve adherence rates and inhaler technique, resulting in improved asthma control.

## Aerosol Therapy—Why we are Failing our Patients

Inhaled corticosteroids have for many years become the backbone of chronic asthma management in children (1–3). They suppress the persistent eosinophilic airway inflammation reducing the propensity for the airways to bronchoconstrict in response to stimuli. This, thereby, reduces both day-to-day symptoms and the risk of a future asthma attack while also improving lung function. Inhaled corticosteroid therapy (ICS) allows the delivery of steroid directly onto the airway wall surface potentially giving great beneficial effect while minimizing side effects.

To get the maximum benefit from ICS they firstly they need to be taken properly with correct inhaler technique so that steroid is optimally delivered to the airway and, secondly, they need to be taken regularly each day even when the child appears well, i.e., with good adherence.

There is now ample evidence that the two main reasons why asthma remains poorly controlled in so many children is that they can't/don't take their ICS inhaler correctly (incorrect inhaler technique) and / or that they simply do not take the ICS on a regular basis (poor adherence) (4–6).

In this paper we review both aspects of basic asthma care. We address each of these two fundamental aspects of asthma care (incorrect inhaler technique, poor adherence to ICS) through key questions such as;

1. how frequent and how important is the problem?
2. how can the problem be identified and addressed?
3. what evidence is there that addressing the problem results in clinical benefit?

The prominent reason for asthma control remaining poor for many children is that the ICS does not reach the targeted airways. This occurs if the ICS medication in not taken (poor adherence) and /or if there is incorrect and poor inhaler technique. No matter how effective an asthma medication has been shown to be if it is not delivered it cannot be effective. In addition, poor inhaler technique with poor drug delivery incurs the medication cost but with a reduced potential clinical benefit in terms of reduced asthma attacks or asthma control.

## Inhaler Technique

The optimal use of medications for asthma is complicated by the fact that most asthma medicines are delivered via inhalation. Children and parents are therefore required to not only understand what the medications do and when to use them but they need also to acquire and master the skills to correctly use the inhalers. An additional problem is that there are a wide variety of different inhaler devices available each with their own specific instruction set for optimal use (7).

In general, pressurized metered dose inhalers (pMDI) require firstly a deep expiration, the MDI is then placed into mouth with lips providing a seal. This is followed by starting a long slow inhalation while pressing the MDI once inspiration has started, followed by a breath hold for about 5 s. The hand-breath coordination is difficult for the majority of children and therefore breath activated devices can be used. Younger children may require a valved holding chamber (spacer device) with or without a face mask. For pre-school children slow tidal breathing may be used through the spacer while ensuring that there is a good face mask seal. Delivery of inhaled medications to infants and toddlers is often associated with crying and mask rejection and this may not be improved by administration during sleep as many infants simply wake up. Having a mouth soother within the face mask may facilitate useful drug delivery to the lungs (8)

With the dry powder inhalers (DPI), in general, the child should first breathe out, place the DPI mouth piece in the mouth, then take a rapid deep inhalation and breath hold.

### Key Question 1. What Is the Prevalence of Poor Inhaler Technique?

In a landmark systematic review Sanchis et al. looked at all published studies from the last four decades assessing the prevalence of correct inhaler technique in all aged patients with asthma/Chronic Obstructive Pulmonary disease (COPD) (9). They found that despite inhaler device improvements the average percentage of asthma/COPD patients with correct inhaler technique was 31% and that this remained constant for each decade (40–30 years ago, 30–20 years ago, 20–10 years ago and the last decade). Sanchis therefore concluded “*Incorrect inhaler technique is unacceptably frequent and has not improved over the past 40 years, pointing to an urgent need for new approaches to education and drug delivery”* (9).

A similar systematic review has been reported by Gillette et al. who reported specifically on inhaler technique in children with asthma (10). Gillette similarly reported convincing evidence that few children can use their inhalers correctly. For children using a pMDI (± spacer) studies varied between 1 and 58% who had good inhaler technique. For children using a DPI this varied between 0 and 35%.

It is important to note that different assessment tools have been used when deciding whether a patient has correct inhaler technique and there exist myths about how to use inhalers (11). Many studies record the number of steps correctly performed out of the total in the manufacturers' recommendations (12). It is difficult to determine whether such studies were being over stringent with this assessment and some patients who may not have correctly completed each recommended step may nevertheless have delivered a significant dose of medication to the airways. Other studies, while recording against a checklist have grouped inhaler technique into correct, partial and poor—with poor technique suggesting that the assessor thought the child would not likely get much medication if they used this technique all the time (13).

### Key Question 2. What Are the Common Inhaler Technique Errors That Are Made?

Critical inhaler handling errors are device specific and occur when a patient performs an error or errors that is/are likely to significantly impair drug delivery to the airway on most or all occasions and have been comprehensively described by Price et al. (14). Table 1 lists the errors in a simplified fashion (14).

**Table 1:** Summaries typical errors in inhaler technique for both metered dose inhalers (MDI, with/without spacers) and dry powder inhalers (DPI) modified from Price et al. (14).

**Type of devices**	**Common critical errors identified (each device has specific steps for correct inhalation—check the device literature insert)**
Metered dose inhaler (MDI) without a spacer	• Fails to shake MDI before dose • Doesn't remove the cap • Doesn't place correctly into the mouth or seal mouth • Timing of actuation incorrect or failure to actuate • Inhaling too quickly, to slowly or not inhaling at all • Not holding breath after inhalation • Doesn't know when to order a new MDI
Metered dose inhaler (MDI) with a spacer	• Failure to remove the inhaler cap or shake the MDI before use • Fails to ensure the MDI is correctly inserted into the spacer device with a tight seal • Doesn't use the device with the inhaler upright at 90° • Actuates more than one dose into the spacer at the same time • Actuates the dose into the spacer before the mouthpiece is placed in the mouth or mask over the face • Fails to make sure there is a tight seal with their lips around the mouthpiece, or on the face if with a mask • Uses the spacer with no dose actuated • Inhales too quickly • If using tidal breathing; inhales too quickly or uses shallow breaths • Failure to keep the head upright • Doesn't prime the spacer correctly • Keeps the spacer in a plastic bag which increases static • Doesn't wash the spacer enough or too often and doesn't leave to air dry • Doesn't replace the spacer if faulty or damaged or as recommended
Dry powder inhalers (DPI)	• Not correctly removing the device cap if appropriate, sliding the cap back fully if necessary or shaking the DPI during preparation • Not fully twisting to release the dose • When the DPI is primed turning it upside down • Holding the device in the mouth while priming • Breathing into the device • Failing to ensure a tight seal with the lips around the mouthpiece • Not breathing out fully before inhalation • Inhaling through the nose • Not keeping head upright with chin tilted slightly upwards • Not taking a hard fast and full inhalation for as long as the patient can achieve • Not holding their breath for >5 s • Not priming before the next dose if more than one being taken • Not knowing when the DPI needs reordered

### Key Question 3. Does It Matter If a Child's Inhaler Technique Is Not Perfect?

Overall (children & adults, asthma & COPD) there is evidence that poor inhaler technique is associated with adverse outcomes. Kocks et al. recently reported a systematic review of studies linking inhaler technique, assessment of errors, and disease outcomes (the majority studies being in asthmatics) (15). Although the 12 asthma studies had various outcomes most studies reported that inhalation errors were associated with worse disease outcomes. They also noted that those patients who had an improved inhaler technique (reduced errors) over time had improved outcomes. They concluded “*These findings suggest that time invested by healthcare professionals is vital to improving inhalation technique in asthma and COPD patients to improve health outcomes.”*

Gillette et al. in their systematic review (see above) also addressed this key question specifically for children with asthma (10). Most of the studies they identified had inhaler technique assessment and training embedded within larger intervention studies and only a few of the 12 studies directly examined how inhaler technique related to asthma outcome. They concluded that there was insufficient evidence in this area and that future research should examine whether improved inhaler technique results in better asthma control, increased quality of life and reduced asthma attacks.

Given that we know that regular use of ICS is overall beneficial for children's asthma control it is only logical to assume that poor delivery of the ICS to the airway target is a bad thing.

### Key Question 4. Why Do Asthmatics Remain Having Poor Inhaler Technique?

It would appear that asthma services do not afford the time to properly train children with asthma to master how to use their inhalers. Simply prescribing an inhaler for delivery of ICS with no instructions will mean that it depends on how well the child/parents can read instructions in the medication leaflet and carry out the correct action. Demonstrating correct inhaler technique to the child/parent is at least one small step better. The commonly used brief instruction only is almost certainly why we are currently still in the situation of only approximately one in three patients being able to correctly use their inhaler (9).

Many asthma patients are managed by non-asthma specialists who, themselves, may not know how to use an inhaler and definitely don't know how to teach correct inhaler use. Indeed, Plaza et al. (16) showed that very few clinicians who have to manage asthma patients can correctly use inhalers. They concluded that “*Health Care Professionals demonstrated inadequate knowledge of the proper use of inhalers. The poor understanding of the correct use of these devices may prevent these professionals from being able to adequately assess and teach proper inhalation techniques to their patients”* (16).

Most asthma management guidelines (1, 2) strongly recommend that inhaler technique should be assessed at every opportunity however there is evidence that this is often not done (17). Suggested barriers to checking/correcting inhaler technique include insufficient clinic time, limited clinician training and knowledge on inhalers and lack of demonstration supplies (18). In addition, health care professionals may ask children/parents about their ability to use their inhaler and use the reply confidence as a proxy to determine whether education is needed. However, it is known that children and especially parents overestimate inhaler skills. In one study it was found that while two thirds of children reported confidence with correct inhaler technique 2% could actually demonstrate correct technique (19, 20).

Time invested in ensuring clinicians (doctors, asthma nurses, pharmacists, respiratory physiologists) are themselves adequately trained in both inhaler technique and how to teach children inhaler technique is important. In addition, time invested in proper training of children to mastery of inhaler technique is important. It is worth comparing the training that the child and family receive when a newly diagnosed diabetic is being trained on how to administer insulin—it would be regarded as negligent to simply show the child/family how to give the insulin and to expect them to be able to manage satisfactorily at home.

### Key Question 5. What Educational Interventions Help With the Mastery of Inhaler Technique?

Asthma guidelines consistently recommend that clinicians check inhaler technique of patients at every opportunity but what is not clear is how can clinicians best ensure patients have good inhaler technique and what interventions they should take if they find a patient with poor technique.

**Figure 1:**
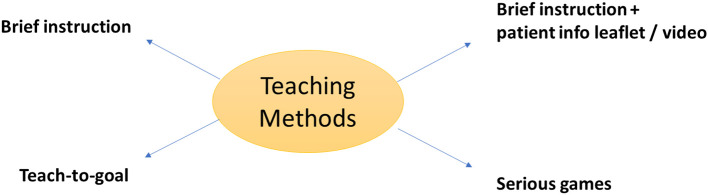
Shows the teaching themes identified that may be suitable for use at an asthma clinic.

We carried out a quick informal scoping review to find out various teaching methods that have been used at teaching inhaler technique. We explored Medline database using the terms “asthma”, and “inhaler technique” and “educational intervention” or “teaching method.” We grouped the inhaler teaching methods into the themes in Figure 1.

Some typical educational interventions identified include either [1] brief instruction, [2] brief instruction plus written instructions with diagrams or an instructional video to be reviewed by the patient (often via a website link or YouTube). Technique demonstration along with verbal instruction has been shown to be superior to verbal instruction alone (21). Poor inhaler technique can remain even after inhalation instruction (22). Shields et al. using Mobile Remote Video Directly Observed Therapy (MDOT) observed that 80% of children under investigation for Difficult to Treat Asthma (DTA) were making an important inhaler mistake during the first week of observation and it took a full 3 weeks of feedback before they could confirm correct inhaler technique. Interestingly all children with DTA improved over the 6 weeks MDOT during which both inhaler technique and adherence to ICS was assured (23).

A variety of helpful tools are available to help with training children on inhaler technique. Examples for demonstration include the Turbohaler Trainer Whistle (®AstraZeneca) which has a whistle if the child does a deep inspiration. An interesting device, the Trainhaler (®Clement Clarke International) has been designed to improve the patient hand lung coordination along with slow and deep inhalation. When the patient inhales through the device they hear the whistle sound. Once the whistling first starts this signals for the patient to press (actuate) the pMDI and the patient is encouraged to keep inspiring to keep the whistle sound going. Ammari et al. have reported that using the Trainhaler increased inhaler technique mastery in children and even resulted in an improvement in subsequent asthma control (24).

A practical solution that seems to be gaining favor is the use of the Teach to Goal method and which is effectively recommended in the new GINA 2019 asthma guidelines (2, 12, 25, 26).

**Figure 2:**
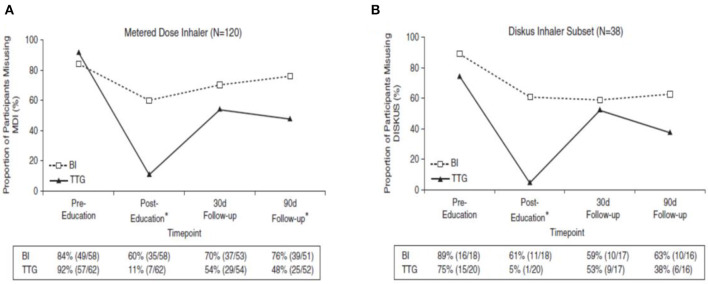
The figure shows the proportion of patients making an inhaler error before and after an educational event comparing brief instruction (BI) with teach to goal (TTG) for **(A)** the metered dose inhaler (MDI) and **(B)** the Diskus dry powder inhaler. Adapted with permission of the American Thoracic Society. Copyright © 2020 American Thoracic Society. All rights reserved. Cite: Press VG, Arora VM, Trela KC, Adhikari R, Zadravecz FJ, Liao C, et al. Effectiveness of interventions to teach metered dose and diskus inhaler technique. *Ann Am Thorac Soc*. (2016) 13:816–24. Annals of the American Thoracic Society is an official journal of the American Thoracic Society. Readers are encouraged to read the entire article for the correct context at [Website Link: doi: 10.1513/AnnalsATS.201509-603OC]. The authors, editors, and The American Thoracic Society are not responsible for errors or omissions in adaptations.

In Teach to Goal (TTG) the child is not just given instruction but first asked to explain back to the educator the key steps in the inhaler technique and why they are needed. This is then followed by the child demonstrating back (or teaching back) to the instructor the use of the inhaler. The instructor provides corrective actions and uses this iterative demonstrate back process until the child has mastered the correct inhaler technique. On three separate occasions during the educational session the child (and/or parent) is asked to retrieve from memory why each step is needed and then demonstrate the correct technique back. The beneficial effects of implementing the TTG approach are illustrated in a randomized controlled trial in adults comparing brief inhaler instruction education with brief instruction plus Teach to Goal (12). This study reported a baseline poor inhaler technique close to 90%. Immediately following the educational session more patients who had been exposed to TTG (90%) demonstrated correct inhaler technique compared with 40% in the brief instruction group. Unfortunately this benefit was not maintained by 30 days (Figure 2) (12).

The TTG approach has also been shown to be feasible within the outpatient clinic setting and only adds 4–5 min to the consultation duration (12). Although TTG took three times longer than BI (mean of 6.3 vs. 2 min) to teach inhaler technique. In addition, it is beneficial for children. Volerman et al. recently reported that while inhaler misuse was highly prevalent in minority children in the USA, using TTG was highly effective at improving all aspects of inhaler technique in the short term (27).

The TTG methodology has even been used remotely using videoconferencing (26). Given that the beneficial effects of using TTG for ensuring that asthmatics have mastery of their inhaler technique may not be long lived Takaku et al. assessed how many teaching sessions were needed before adult patients had completely correct inhaler technique (28). They reviewed patients' inhaler technique every 2 weeks and until no further improvements were being made. For all inhaler devices they found that it took at least three instructional sessions were required and each patient had correct technique.

It is not hard to see how patients are being failed when looking at the quality of assessment and education regarding inhaler technique being delivered in time constrained clinics. With realistic review template slots and more frequent reviews there would be enough time to choose the best delivery device for the individual patient and assess inhaler technique using Teach to Goal. There is also a need for the staff delivering clinics and training to be competent in every device they recommend or prescribe to patients, they should not just be handing over a leaflet and directing the patient to videos, but instead ensuring that the patient is confident in knowing exactly what to do with the device before they leave the clinical area.

## Summary Bullet Points—Inhaler Technique

- Poor or incorrect inhaler technique is very common and is associated with poor asthma control in both adults and children.
- Clinicians who at some time have to prescribe asthma inhalers should be properly trained in both their use and how to teach children to use inhalers correctly.
- Time is well invested in ensuring asthma children have mastered the use of their inhalers.
- Using Teach to Goal approach is one proven way that can easily be implemented during an asthma educational session.
- Unfortunately the ability to demonstrate correct inhaler technique is a skill that can be lost over time and multiple (e.g., 2–3) teaching sessions may be needed.

## Adherence

Poor asthma control can be attributed to an incorrect inhaler technique as previously described, but across all age groups adherence to therapy remains a significant, modifiable barrier to effective treatment. Adherence has been defined by the World Health Organization as “the extent to which a person's behavior—taking medication, following a diet, and/or executing lifestyle changes, corresponds with agreed recommendations from a health care provider” and in practice is the percentage of prescribed doses actually taken by the patient.

Adherence to asthma treatments such as inhaled corticosteroids is crucial to gaining optimal disease control given that it is undoubtedly a highly effective method of reducing bronchial hyper-responsiveness. Despite it being very effective, adherence has been shown to be poor across all age groups, at least in part explained by the need for 4–6 weeks of regular treatment to reach its peak effect.

We will explore the impact of poor adherence to asthma treatment, reasons why children might not take their treatment and the methods by which adherence can be accurately measured and optimized.

### Key Question 1. What Rate of Adherence Is Necessary for Optimal Asthma Control and What Are the Average Rates of Adherence When Recorded Objectively?

An adult study of prescription records found that a minimum adherence to inhaled corticosteroids (ICS) of 75% was associated with a significant reduction in the incidence of asthma exacerbations (29), but despite this, objective adherence studies show that this is often not achieved.

A meta-analysis found that the adherence to electronically monitored preventer inhalers in children was <50% in half of the studies identified and all but one had a rate of <75%. Risk factors for poor adherence included being an older child and having a low socioeconomic background (30). Developed countries such as the UK and the Netherlands demonstrated better adherence although this was still <75% in most cases (30) and is probably higher than the true value outside of a study setting. Table 2 shows an updated summary of the average adherence rates from the control groups of studies using electronic monitors.

**Table 2:** Average adherence rates from studies using electronic monitoring.

**Study**	**Number of subjects**	**Age (years)**	**Duration (months)**	**Control group adherence rate (%)**
Gibson et al. (31)	26	1.5–5	2	71
Milgrom et al. (32)	24	8–12	3	Median 58
Bender et al. (33)	27	7–12	6	50
McQuaid et al. (34)	106	8–16	1	48
O'Connor et al. (35)	16	8–18	3	54
Burgess et al. (36)	21	1.5–7	3	Median 65
Bender and Zhang (37)	104	8–18	4	40
Burgess et al. (38)	51	1.5–7	1	Median 71
Jentzsch et al. (39)	102	5–14	12	52
McNally et al. (40)	63	5–17	1	34
Burgess et al. (41)	26	6–14	4	58
Celano et al. (42)	1433	6–11	12	57
Nikander et al. (43)	115	5–10	18	73
Jentzsch et al. (44)	102	5–14	12	47
Feldman et al. (45)	102	7–15	1.5	28
Klok et al. (46)	93	2–6	3	Median 92
Vasbinder et al. (47)	87	2–11	3	49
Duncan et al. (48)	48	9–15	5	49
Chan et al. (49)	220	6–15	6	30
Vasbinder et al. (50)	209	4–11	12	57
Morton et al. (51)	77	6–16	12	49
Kenyon et al. (52)	41	2–13	1	32
**Mean adherence from 22 studies** **=** **51%**				

*Rates are the mean (or median) rate of the control groups*.

### Key Question 2. What Are the Consequences of Poor Adherence in Children With Asthma?

Adherence in asthma is universally poor despite many of the treatments being highly effective and uncontrolled disease having a significant morbidity and mortality. The danger of poor asthma control and adherence to treatment was underlined by the 2014 National Review of Asthma Deaths (NRAD) in the UK in which 38% of those who died had been prescribed <25% of their ICS dose, and 80% had been prescribed less than their full dose in the preceding year (53).

Drug holidays and alterations in dose across the week for convenience can result in suboptimal dosing and be associated with morbidity (44), leading to more exacerbations and more courses of oral steroids (37). Poor ICS adherence increases sputum eosinophil counts and reduces FEV_1_ (54), all of which have a negative impact on quality of life (55). Uncontrolled disease is associated with an increase in healthcare use via General Practitioners, Emergency Department attendances and other hospital visits (56, 57), all of which can be disruptive and detrimental to children's social and educational development.

### Key Question 3. What Methods of Adherence Monitoring Are Available and Are They Reliable?

#### Self-Reporting

The easiest and most commonly used way to assess adherence is to ask the child and/or their carers during hospital visits. Whilst it is expected that many patients over report their adherence, clinicians could only identify 55% patients who were inaccurately self-reporting when assessed by electronic monitoring (38). The propensity for patients to overestimate their adherence is called a social desirability bias and this is usually driven by a desire to be looked upon favorably by clinicians or carers if children take their medication independently.

A less confrontational method of self-reporting can be achieved with a questionnaire filled out prior to their consultation. Despite this perceived advantage, these questionnaires remain subjective and their accuracy is also affected by social desirability bias, poor recall and a generalization of behavior rather than recalling actual events. These challenges were demonstrated in an American study of perceived adherence which was 85% compared to 25% after objective measurement (58).

It might be thought that poor recall and reporting generalized rather than actual adherence events would be improved by a daily diary of medication. With the increasing use of mobile technology, completing these diaries can be facilitated via apps and more accessible formats but studies show that these are also inaccurate and it is likely that the challenges to electronic records are likely to be similar. Whilst some patients will diligently fill out diaries, this behavior and therefore the adherence data collected, favors motivated patients and their families and would not be likely to reflect the population as a whole. This is supported by studies showing that diaries overestimate adherence (31) and the suspicion that in some patients' whole diaries are filled in on a single day.

Phone calls to discuss the medication taken in the preceding 24 h is another approach but this is labor intensive. Whilst it may improve adherence on contact days, such effects are likely to be short-lived and subject to the Hawthorne effect, a phenomenon describing the increase in adherence subsequent to a period of monitoring followed by a waning of this improvement once the observation ends back to usual levels.

#### Pharmacy Dispensing Records

Prescription records detailing the number of times a medication was dispensed is an objective measure of adherence but this has many challenges. Importantly it only tells you about the number of available doses and not whether they were delivered (or how they were delivered) and therefore in general has a tendency to overestimate adherence (59). Furthermore, it relies upon medication not being dispensed from sources other than primary care and may miss those collected following hospital admissions or clinic visits. Despite these shortcomings it does give some idea of a patient's adherence in a quick and inexpensive way and is useful at identifying poorly adherent patients who have collected medications in quantities far less than would be needed to meet their requirements.

#### Canister Weights and Dose Counters

Weighing inhaler devices and documenting dose counters can be used to objectively measure adherence although neither provide data on the timing or effectiveness of the doses. Whilst these measurements can be useful as a guide to adherence in some patients, it can also be manipulated by others leading to an overestimation of adherence. Doses can be “dumped” prior to assessment, by which the patient actuates the device multiple times to make the counters look more favorable. This has been demonstrated in between 18 and 30% of asthmatics (60, 61).

#### Electronic Monitoring

**Figure 3:**
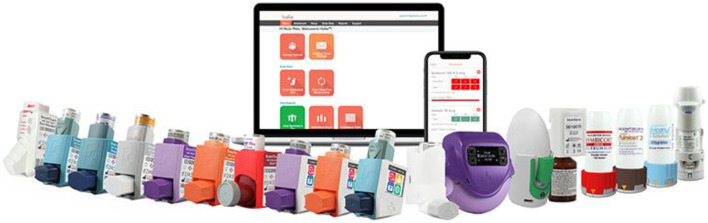
Electronic monitoring devices manufactured by Adherium ltd. (www.adherium.com).

Electronic monitoring is an objective method of assessing adherence to inhaler and nebuliser therapy as it enables dose measurements coupled with a timeline in order to provide an accurate reflection of medication usage (Figure 3). Early problems with devices malfunctioning and limited battery life affected accuracy, but with technological improvements they are now proving to be a valuable tool in reliably monitoring adherence although their price precludes their use in some settings. Electronic devices can either be added to, or increasingly incorporated into, inhaler and nebuliser devices. They provide data by being downloaded or sent wirelessly in real-time to provide accurate adherence monitoring outside of hospital visits (62). Furthermore, additional features such as audio-visual reminders, have been shown to increase adherence from 30 to 84% in children taking ICS (49). Whilst the data is robust, the effectiveness of dose delivery with a good inhaler technique cannot be measured, neither can it meet the challenge of identifying those patients who actuate devices regularly without inhaling any drug. Within cystic fibrosis, however, electronic monitors on nebulisers additionally measure the duration of dose delivery and can therefore record if the whole dose is delivered (63), a feature that has not as yet been incorporated into asthma monitoring.

### Key Question 4. What Are the Barriers to Optimal Adherence in Children With Asthma?

#### Intentional Barriers

Intentional barriers to adherence are conscious decisions not to adhere to treatment and may be difficult to identify and even more challenging to successfully address. Having a relationship with patients enabling them to feel comfortable discussing their worries is pivotal, but this may be very difficult to achieve.

Taking a medication each day to maintain health means that for the majority we are asking them to do so whilst feeling well. The perceived benefit of treatment has a significant effect on adherence, particularly if that medication takes up to 6 weeks to show an effect as is the case with ICS, is difficult to administer or is deemed unpleasant (64).

A frequently cited worry with asthma treatment is the effect of steroids on growth and on the immune system. Similarly the regular nature of effective asthma treatment causes concern with worries that it will mask rather than treat the problem and that the body will eventually become used to it, a belief that affects long term adherence to effective treatment. Addressing these often entrenched beliefs is pivotal to improving adherence but this can take time and may not be possible within the constraints of a single clinic visit. One study of ICS adherence showed the highest levels among those with parents who had a positive view of ICS prior to commencing treatment (63). Understanding our patients as individuals, focusing on medication education, responding to the educational abilities of our families and utilizing the skills and resources of asthma nurses are all vital to enhancing adherence to asthma therapy. Despite this, in many countries the financial constraints of purchasing treatment ultimately determines adherence, and the maintenance of health in chronic conditions with expensive therapies being required every day for patients who feel well most of the time may not be prioritized and thus will adversely affect the poorest in society.

#### Unintentional Barriers

Unintentional barriers are just as difficult as intentional barriers to identify and improve, and it is likely that they have a greater impact on adherence.

Patients' and carers' understanding of treatment regimens can greatly affect asthma treatment and the effect of an educational intervention alone remains uncertain (65). In families with learning difficulties this can be even more challenging to address. Expecting a regular medication routine in a chaotic household is difficult, with “simply forgetting” being a common reason cited for inadvertently missing treatment. Not incorporating medication into a daily routine is a frequent explanation for poor adherence, as is running out of time (38), a factor that is enhanced if numerous medications or time-consuming therapies are being prescribed. In some families, adherence can be improved during the school week when there is more routine or in some patients who can have medications administered at school. In adults with asthma, a text message reminder to take medication did improve adherence (66) but the long-term value of such a system, particularly in pediatric care, remains unproven.

Psychosocial factors are known to affect disease outcomes and should also be assessed within the multi-disciplinary team (67). Children with asthma are known to suffer with increased emotional and behavioral problems and emotional difficulties for parents and caregivers may affect their ability to care for their child. There is evidence that factors such as depression can play a role in adherence (68) to medication and treatment of these factors are important to improve asthma control. The age of children can affect their adherence. Within the pediatric population, younger children who have medication administered by an adult show the highest levels of adherence (69). Despite this there can be many challenges, and oppositional behavior makes delivering medication regularly and effectively very difficult (38). Fitting all the doses into a young child's routine, particularly if they fall asleep prior to the evening dose, can also affect adherence.

Adolescence brings similar adherence challenges but at this age it is often the desire not to be different to peers, and the social barrier to taking medication that highlights them as different, that can make treatment challenging (70). Depression has been shown to adversely affect adherence in children with cystic fibrosis (71) and this likely to be similar in asthma. Although it is suspected that parents with mental health problems are likely to have children with poorer adherence, this association has not yet been elicited (30). Engaging adolescents and gaining their trust is pivotal to understanding what barriers they have to taking treatment regularly and how adherence can be improved.

### Key Question 5. Can Electronic Monitoring With Feedback Improve Adherence Rates and Outcomes in Asthma?

Electronic monitors with structured feedback of adherence information that is then delivered by a member of the multidisciplinary team has been shown to significantly increase adherence to treatment in adults with COPD (60) and adults with asthma (72), although in the latter group this did not translate into better asthma control. Structured feedback of electronic data to patients has also been shown to significantly improve adherence in children (41, 51, 73). A UK study of children with poorly controlled asthma had significantly better adherence with electronic monitors and feedback compared to monitoring alone. In addition the higher adherence rates were maintained throughout the 12 month follow-up period. Although this did not translate into better Asthma Control Questionnaire (ACQ) scores, there were significantly less hospital admissions and courses of oral steroids in the group receiving feedback (51). A study in New Zealand using electronic monitoring with reminders but no feedback demonstrated a significant improvement in adherence rates (detailed above), although this effect waned over time. This study did not demonstrate an improvement in clinical measures (49). The combination of these results would suggest that regular structured feedback helps to maintain optimal adherence rates and this translates into clinical benefits for the patient.

It is likely, with the advent of telehealth, that the remote access of monitoring data by clinicians, will become more commonplace. Although the successful role of smart technology is yet to be proven in the literature (74), and asthma technology has focused more on the delivery of educational and self-health programmes, it is anticipated that electronic monitoring and instant feedback both to the patient and their doctor will allow continuous assessment of adherence outside of the clinic setting.

### Key Question 6. Can Other Methods of Inhaler Administration Improve Adherence?

Electronic monitoring is costly, time-consuming and there are significant supply issues with smart inhalers. Moreover, despite the knowledge of being monitored many children and teenagers remain non-adherent. Recent data suggests that intermittent therapy with combination inhalers as advocated in the GINA 2019 strategy document may be efficacious in preventing asthma attacks (2). The data does suggest that regular daily anti-inflammatory medication may be best but it appears that this cannot be achieved in the majority of patients. Here intermittent therapy could be supported and monitored by health care practitioners.

## Summary—Adherence

- Subjective methods of adherence monitoring are inaccurate and underestimate rates of non-adherence
- When electronically measured, average adherence rates are around 50%
- Barriers to adherence can be intentional and unintentional, and both sides need to be addressed to effectively improve adherence rates
- Electronic adherence monitoring can accurately measure rates, enabling data to be fed back to patients and families to facilitate change in behavior to increase adherence.

## Smart Technology to Optimize Asthma Control

The challenges to achieving optimal asthma control include adequate education, correct inhaler technique and good adherence. Adolescence poses further difficulties as children transition to young adulthood and become more independent, many taking ownership of their medication for the first time, whilst also social pressures increase and life becomes less routine. It is not surprising, therefore, that adolescents have among the lowest rates of adherence and asthma control (30), yet most asthma services are aimed at children or adults and therefore do not recognize or adapt to the developmental changes at this age. Asthma care now centers around a partnership model with national and international guidelines advocating the routine use of self-management education. This is aimed at incorporating education, self-monitoring of symptoms and/or peak flow readings, regular medication reviews and emergency plans (75, 76). Engaging all children and their families in managing their own asthma, and in particular adolescents who often do not respond to usual interventions, is a challenge for us all.

In 2015, 73% of US and 80% of Australian adolescents used a smartphone (77), with numbers likely to continue to increase worldwide as retail prices fall. Smartphones are accessible and convenient. Importantly, they have the capacity to rapidly adapt to digital advancements in health services, including access to apps that deliver health care in a non-confrontational manner. These are a valuable resource in addressing asthma management in pediatrics. Health apps can be used by carers, but in particular by older children and adolescents, who are assuming greater responsibilities for their health. Apps can offer personalized medicine with the economic benefit of this all happening remotely, negating the need for as many expensive and often inconvenient hospital visits.

For over 10 years, mobile phone technology has been used in the management of chronic disease with applications such as appointment reminders and short message service (SMS) prompts to take medication (78, 79). Within asthma alone, web-based programmes have also been used to facilitate decision-making via self-help algorithms. These are all aimed at self-management, the process by which patients take responsibility for their condition, actively participate in their care and engage in the emotional management of their disease. This is achieved by education and empowerment, encouraging patients to recognize and respond to symptoms and to adhere to preventative strategies. Engaging patients in self-management processes is challenging but e-health offers a flexible forum rather than relying upon traditional clinic visits that are often too short, too infrequent and too daunting. Sleath et al. found that during one third of asthma consultations preventer medications were not discussed, fears and concerns about treatment were mentioned in 1%, and how well treatment was working in only 12% of clinic contacts (80). Shortcomings in our hospital discussions regarding the important issues that affect asthma control and medication adherence is detrimental to improving the lives of patients with asthma, but in particular those with the poorest disease control.

### Key Question 1. What Smart Inhalers and Apps Are There Available for Use in Health Systems/Commercially for Children's Asthma?

#### Apps

In 2017, a review found 38 publicly available “well adopted” smartphone asthma self-health apps. Ninety-five percentage were free to download and the majority had been developed by private companies and independent developers with only a small proportion of those available being aimed at children or young people (81). Asthma apps enable the dissemination of self-management programmes at a population level. Health apps are becoming more popular and the number available continues to increase, although their quality remains more difficult to assess. A 2013 Cochrane review of asthma self-management apps aimed to assess feasibility, effectiveness and cost-effectiveness in terms of patient-reported measures of asthma control, quality of life and frequency of healthcare visits, as well as secondary outcomes such as lung function, adherence, health economics and patient satisfaction. Unfortunately they were not able to draw any definitive conclusions as only two randomized controlled trials were identified, and they could not be compared due to baseline heterogeneity (74). Although challenging, it is vital to keep up-to-date with the apps available to our patients and to be able to recommend the most useful and advise against others where necessary. Huckvale et al. (81) found that even though the number of asthma apps are increasing rapidly, newer apps are often not better than older ones at providing information and self-management tools.

There is a desire from both patients and clinicians to move asthma care in the direction of e-health. In 2015 Asthma UK asked if patients would want an electronic health system that monitored their symptoms, and nearly 75% said yes. Furthermore, over three quarters of healthcare professionals thought that an electronic system for symptom monitoring and generating an asthma plan would be beneficial (myAirCoach, Asthma UK). Carpenter et al. asked adolescents what they wanted from an asthma app and their priorities included medication and appointment reminders and an accessible asthma management plan with identified triggers, symptoms, and emergency information (82). The emergency asthma plan is often poorly understood, reviewed sporadically and poorly implemented. The incorporation of this into an app was rated highly in Burbank's adolescent study, with 93% reporting that they would adhere better to plans accessed via an app (83) whilst Odom showed that they are easier to follow (84) and Ramsey that they are preferable to a written plan (85).

Barriers to effective asthma control are numerous and include communication difficulties between patients, their families and care providers. Children and adolescents are often reluctant to be seen as different to peers and may try to ignore their asthma, disengaging with management and becoming isolated from families, peer groups and medical staff. App communication streams between care givers and patients adapt to the needs of those who may not want to discuss things directly during clinic visits. Eighty-eight percentage in adolescents in Roberts' study felt happy sending information to medical teams via an app (86) and Ramsey found that 75% were happy for teams to have full access and to contact them if concerns arose, deeming it a way of avoiding hospital visits (85).

Increasing asthma education to facilitate effective self-management has been proven to improve children and adolescents' lung function, feelings of self-control. It has also been proven to reduce absenteeism from school, the number of days with restricted activity and the number of visits to an Emergency Department. Apps provide a forum for asthma education that can be tailored to a patient's age and be personalized to their health needs (87). Despite these advantages, evidence to support their effectiveness remain sparse and there is yet to be demonstrated a significant long-term benefit. Alaquran did not identify any randomized controlled trials in their meta-analysis, with the studies included assessing effects after only a week of using the app and not having a follow up period longer than 8 weeks. They concluded that there was a potential benefit but long-term benefits remained unproven (77). The Cochrane review did not identify any studies of adolescents and therefore could not support their use in adolescence (74).

#### Smart Inhalers

Smart inhalers use a smartphone's Bluetooth connection to collect data via a health app or a website that can be then accessed by the patient and their asthma team. Monitoring devices can be incorporated into the inhaler or can come separately and then be attached. In addition to measuring adherence, smart inhalers can provide audio-visual reminders to take medication and can contain pressure sensors that can analyze the efficiency of actuation. Smartinhalers have been demonstrated to be highly accurate *in vitro* (62, 88) and a Medtech innovation briefing has been developed by the National Institute for Health and Care Excellence (NICE) to support their use (89). As discussed previously, smart inhalers track inhaler usage, giving objective adherence data that can then be reviewed with the patient and have been shown to improve clinical outcomes (51).

### Key Question 2. What Is the Future Direction of Digital Healthcare for Asthma Care in Pediatrics?

Pediatric asthma is a UK National Health Service (NHS) 10 year priority, and innovative approaches to managing the increasing number of children with moderate to severe asthma in specialty pediatric units is required. Technological advances are changing the face of medicine, making healthcare more personalized, more accessible and often more acceptable to patients. NRAD identified many shortcomings in the care of asthma in the UK. It made recommendations for improvements including improving risk stratification, identifying patients who have been prescribed too few or too many inhalers, improving medical records and follow up pathways, and facilitating self-management systems (53). All these areas can be targets of digital intervention and improvement.

Medical sensors are being designed and trialed within asthma to add additional medical data streams. In 2010 Boner measured nocturnal wheeze in asthmatic children and found that 57% of children with good lung function and few symptoms had significant night-time wheezing (90). Devices have also been used to continuously monitor heart rate and respiratory rate and to count coughs. Digital techniques and the potential to develop artificial intelligence algorithms are likely to provide the ability to incorporate the interpretation of these signals provide an emergency warning system. GPS data also allows this all to be interpreted along with environmental information measuring conditions that can impact on asthma including air pollution and humidity. This more detailed assessment could be used to risk stratify patients and to identify those who may develop a serious exacerbation, alerting both the patient and their clinician. Sensors can be wearable but attempts are being made to also incorporate them into clothing (91).

Safer prescribing is vital and improving electronic health records and enabling prescription records to highlight under or over prescribing of asthma medication, a proxy measure of adherence, is an NRAD recommendation to avoid asthma deaths (53). Electronic alerts informing healthcare professionals of attendances at Emergency Departments would also identify patients in need of closer monitoring, a system that would be part of a more sophisticated electronic health record promoting greater communication across all teams.

The increased use of health technology will inevitably bring its challenges. In many countries the price of inhalers already prohibits their use, but with a dependence on smart technology this puts an even greater financial burden on health systems and individuals that may not be sustainable. Even in wealthy countries, plans need to be made to meet these increased short-term costs whilst determining what systems are effective, sustainable and where priorities lie. Data security and information governance are additional concerns with large amounts of data being collected electronically. Plans need to be made regarding how data is stored and integrated into electronic health records, and to determine who owns it.

Asthma management is ever changing and it is likely that digital technologies will be pivotal in these advancements. Whilst change must be embraced, it is essential that we support research, appraise developments and promote evidence-based technologies, ultimately encouraging clinical partnerships with our patients that empower them to successfully self-manage, yet always demanding value for money and safe data protection.

## Summary—Smart Technology

- Smart technology enables patients and families to engage with their asthma management via online and mobile technology.
- Asthma apps are available to measure adherence, monitor symptoms, predict exacerbations and provide ongoing asthma education to patients. The information from the apps can be used directly by patients and shared with healthcare professionals to improve asthma control.
- Asthma apps are popular amongst adolescents, a group in whom adherence rates and asthma control is often sub-optimal.

## Author Contributions

MS devised the concept of the article and is an author. RM, HE, VC, and LK all wrote sections of the article. All authors have read and approved the final manuscript submitted.

## Conflict of Interest

The authors declare that the research was conducted in the absence of any commercial or financial relationships that could be construed as a potential conflict of interest.
